# Parvalbumin Interneurons and Perineuronal Nets in the Hippocampus and Retrosplenial Cortex of Adult Male Mice After Early Social Isolation Stress and Perinatal NMDA Receptor Antagonist Treatment

**DOI:** 10.3389/fnsyn.2021.733989

**Published:** 2021-09-22

**Authors:** Patrycja Klimczak, Arianna Rizzo, Esther Castillo-Gómez, Marta Perez-Rando, Yaiza Gramuntell, Marc Beltran, Juan Nacher

**Affiliations:** ^1^Neurobiology Unit, Program in Neurosciences and Institute of Biotechnology and Biomedicine (BIOTECMED), Universitat de València, Burjassot, Spain; ^2^Spanish National Network for Research in Mental Health, Centro de Investigación Biomédica en Red de Salud Mental (CIBERSAM), Madrid, Spain; ^3^Fundación Investigación Hospital Clínico de Valencia, INCLIVA, Valencia, Spain

**Keywords:** schizophrenia, parvalbumin, interneuron, social isolation, early aversive experience

## Abstract

Both early life aversive experiences and intrinsic alterations in early postnatal neurodevelopment are considered predisposing factors for psychiatric disorders, such as schizophrenia. The prefrontal cortex and the hippocampus have protracted postnatal development and are affected in schizophrenic patients. Interestingly, similar alterations have been observed in the retrosplenial cortex (RSC). Studies in patients and animal models of schizophrenia have found alterations in cortical parvalbumin (PV) expressing interneurons, making them good candidates to study the etiopathology of this disorder. Some of the alterations observed in PV+ interneurons may be mediated by perineuronal nets (PNNs), specialized regions of the extracellular matrix, which frequently surround these inhibitory neurons. In this study, we have used a double hit model **(DHM)** combining a single perinatal injection of an NMDAR antagonist (MK801) to disturb early postnatal development and post-weaning social isolation as an early life aversive experience. We have investigated PV expressing interneurons and PNNs in the hippocampus and the RSC of adult male mice, using unbiased stereology. In the CA1, but not in the CA3 region, of the hippocampus, the number of PNNs and PV + PNN+ cells was affected by the **drug** treatment, and a significant decrease of these parameters was observed in the groups of animals that received MK801. The percentage of PNNs surrounding PV+ cells was significantly decreased after treatment in both hippocampal regions; however, the impact of isolation was observed only in CA1, where isolated animals presented lower percentages. In the **RSC**, we observed significant effects of isolation, MK801 and the interaction of both interventions on the studied parameters; in the DHM, we observed a significantly lower number of PV+, PNNs, and PV+PNN+cells when compared to control mice. Similar significant decreases were observed for the groups of animals that were just isolated or treated with MK801. To our knowledge, this is the first report on such alterations in the RSC in an animal model combining neurodevelopmental alterations and aversive experiences during infancy/adolescence. These results show the impact of early-life events on different cortical regions, especially on the structure and plasticity of PV+ neurons and their involvement in the emergence of certain psychiatric disorders.

## Introduction

Schizophrenia is a complex and multifactorial disease with a lifetime risk of about 1% (Schultz et al., 2007), resulting in dramatic cognitive deficits, behavioral and emotional dysfunctions (Harrison, 1999), usually starting from late adolescence and early adulthood (Cannon and Jones, 1996). Although the complex etiology of schizophrenia is still not fully understood, some predisposing factors have already been recognized (Howes et al., 2004), including early life aversive experiences (Corcoran et al., 2003; Read et al., 2005). Some of the most affected brain regions by these early life adverse events, which are also known to be altered in schizophrenia, are the prefrontal cortex (PFC), the amygdala, and the hippocampus (Phillips et al., 2003; McEwen et al., 2016). Interestingly, different reports have also described alterations induced by early life stress in the retrosplenial cortex (RSC; Aksić et al., 2014; Marković et al., 2014), a region within the posterior neocortical system, interconnected with both cortical and subcortical brain networks (Vogt et al., 2004). The RSC is involved in several cognitive tasks, including spatial memory, navigation, object recognition, imagination and planning (Mitchell et al., 2018), and some studies have reported that it undergoes structural and functional alterations in schizophrenic patients (Mitelman et al., 2005; Siemerkus et al., 2012; Wang et al., 2015). However, very little is known about the cellular and molecular modifications underlying these alterations. Interestingly, the RSC is especially vulnerable to the action of N-methyl-D-aspartate receptor (NMDAR) receptor antagonists, which have propsychotic actions and are used frequently in animal models of schizophrenia (Olney et al., 1999).

The etiopathology of schizophrenia is far from being understood, especially the changes at the cellular level that can underly this disorder are only starting to be discovered. However, numerous studies in patients and animal models have found alterations in the subpopulation of parvalbumin (PV) expressing interneurons, particularly in the PFC (Lewis et al., 2012; Gonzalez-Burgos et al., 2015) and the hippocampus (Zhang and Reynolds, 2002; Lodge et al., 2009; Koh et al., 2016). Interestingly, recent reports have also described alterations in this population of inhibitory neurons in rats subjected to maternal deprivation, an environmental risk factor for schizophrenia (Aksic et al., 2021), or in mice with a mutation in a gene associated to this psychiatric disorder (Schmalbach et al., 2015).

PV+ interneurons are fast-spiking GABAergic neurons that provide inhibitory control of cortical and subcortical circuits and regulate the synaptic excitatory tone in different cortical regions (Hu et al., 2014). During postnatal development, they are involved in activity-dependent changes in cortical maturation and the control of critical periods (Reichelt et al., 2019; Carulli and Verhaagen, 2021). These interneurons are also crucial for the maintenance of gamma rhythms and the inhibition/synchronization of pyramidal neurons Hashimoto et al., 2003; Uhlhaas and Singer, 2010; Allen and Monyer, 2015). Some of these changes seem to be mediated by crucial regulators of interneuronal plasticity, such as the components of the perineuronal nets (PNNs), specialized regions of the extracellular matrix, mainly present around the soma and proximal neurites of PV expressing interneurons (Reichelt et al., 2019; Carulli and Verhaagen, 2021). PNNs provide a physical barrier, maintain the local ionic homeostasis, and protect neurons against oxidative stress (Wen et al., 2018; Bosiacki et al., 2019; Testa et al., 2019). They can also interact with ion channels on the cell membrane and control synaptic plasticity by stabilizing recently formed synapses and preventing synaptogenesis (Faissner et al., 2010; Hayani et al., 2018). PNNs also regulate the inhibitory input on PV+ cells and gamma oscillations, which are dependent on these inhibitory neurons (Carceller et al., 2020; Wingert and Sorg, 2021). Interestingly PNNs are remarkably altered in the amygdala, as well as in the entorhinal and PFC of subjects diagnosed with schizophrenia (Pantazopoulos et al., 2010; Mauney et al., 2013; Markota et al., 2014). Consequently, they may play an essential role in the pathophysiology of this disorder (Berretta et al., 2015).

Alterations in other populations of interneurons have also been observed in the brain of schizophrenic patients and in animal models of this mental disorder (Dienel and Lewis, 2019). However, since PNNs are almost exclusively surrounding PV+ cells in the cerebral cortex, we decided to focus our analyses on these fast-spiking interneurons.

Several animal models have been generated to investigate the relationship between early life aversive experiences and the etiology of psychiatric disorders such as schizophrenia (Marcotte et al., 2001; Winship et al., 2019). Most of them include manipulating maternal interaction, disrupting either the quantity or quality of maternal care early in life (Bolton et al., 2017). One of them is the postweaning social isolation model, intended to simulate adverse experiences during early life in humans. This model reproduces some of the alterations observed in schizophrenic patients (Powell et al., 2012; Wang and Fawcett, 2012). This model has been lately combined with experimental interventions during early postnatal life. One of them is the administration of NMDAR antagonists, which alter transiently the latest stages of neocortical development (Makinodan et al., 2012). The perinatal injection of NMDAR antagonists combined with the postweaning social isolation rearing has been used as an animal model for schizophrenia, in which some behavioral, structural and neurochemical traits similar to those found in schizophrenic patients can be found. We have previously shown in mice that the combination of these paradigms in a double hit model (DHM) causes changes in anxiety and locomotor behaviors (Castillo-Gómez et al., 2017) and a decrease in prepulse inhibition of the startle reflex (Garcia-Mompo et al., 2020). In addition, we found that the volume, the structure and connectivity of excitatory and inhibitory neurons, and the expression of molecules related to inhibitory transmission and its plasticity were altered in the PFC and the amygdala (Castillo-Gómez et al., 2017; Garcia-Mompo et al., 2020).

Interestingly, we also observed alterations in the number of PV expressing neurons and PNNs in certain regions of the amygdala and the PFC of DHM mice (Castillo-Gómez et al., 2017; Garcia-Mompo et al., 2020) and PV+ neurons in the PFC of DHM rats (Gilabert-Juan et al., 2013). However, we still do not have information on the impact of this animal model on the PV expressing cells and PNNs of the hippocampus and the RSC of adult mice. Consequently, we have evaluated the effects of the DHM and each of its factors on the number of PV expressing neurons, PNNs, and their co-localization using immunohistochemistry, confocal microscopy, and unbiased stereology.

## Materials and Methods

### Animals and Experimental Treatment

In the present experiment, we used **28** adult male FVB mice. Animals were subjected to a DHM previously described by our laboratory in this mouse strain (Castillo-Gómez et al., 2017). Three-month-old animals (one male and two female mice) were kept in breeding cages under standard light conditions (12 h light/dark cycle) and temperature, with *ad libitum* access to food and water. All mice were housed in the same room, sharing the same controlled environment. To prevent disruptions among mice, pregnant females were housed individually. 7 days after birth (P7), male **pups selected** randomly received a single intraperitoneal injection of MK801 (1 mg/kg solved in NaCl 0.9%, Abcam) or the vehicle solution (0.9% NaCl). The animals were first numbered and tagged, and then half of the numbers were randomly selected for each experimental condition. After the injection, pups were returned to their cages and remained with their mother until the age of weaning (P21). At this age, seven mice from each of the groups (Vehicle or MK801) were randomly selected and housed alone (isolation) in small polycarbonate cages (24 × 14 × 13 cm; Zoonlab-Bioscape) or groups of 3–4 mice (social) in standard-size cages (38 × 16 × 13 cm; Zoonlab-Bioscape) for 10 weeks. Isolated mice could hear and smell other mice, but physical or visual contact with them was not allowed. In this way, the four final experimental groups were determined: Veh-Social (injected with NaCl, and socially reared), Veh-Isolation (injected with NaCl and isolated), MK801- Social (injected with MK801 and socially reared), and MK801-Isolation (injected with MK801 and isolated), which was the DHM group. At P90, the animals were sacrificed. All the experiments were conducted in accordance with the Directive 2010/63/EU of the European Parliament and of the Council of 22 September 2010 on the protection of animals used for scientific purposes and were approved by the Committee on Bioethics of the Universitat de València. Every effort was made to minimize the number of animals used and their suffering.

### Perfusion and Immunohistochemistry

Mice were anesthetized with sodium pentobarbital and transcardially perfused with 4% paraformaldehyde solution for 20 min. All perfusions were performed between 10.00 and 14.00 h. After perfusion, brains were extracted from the skull; their hemispheres were separated and cut into 50 μm-thick coronal sections using a freezing-sliding microtome (LEICA SM2000R, Leica) for immunohistochemical analysis. After cutting, tissue was processed free-floating for fluorescence immunohistochemistry. Selected sections were washed in PBS and then incubated for 1 h in 10% normal donkey serum (NDS; Abcys) in PBS with 0.2% Triton X-100 (PBST; Sigma-Aldrich). Afterward, we incubated them for 48 h at 4°C with a primary polyclonal rabbit IgG anti-PV (1:2,000, Swant) and biotin-conjugated *Wisteria floribunda* agglutinin (1:200, Sigma-Aldrich) to study the expression of PV and PNNs. After washing, sections were incubated for 2 h at room temperature with a secondary donkey anti-rabbit IgG antibody conjugated with AlexaFluor-555 (1:400, Jackson ImmunoResearch) and avidin conjugated with AlexaFluor-647 (1:400, Jackson ImmunoResearch) diluted in PBST and 5% NDS. Finally, sections were washed in PB 0.1 M, mounted on slides, and coverslipped using fluorescence mounting medium (Agilent).

### Quantification of PV Expressing Cells and PNNs

**Figure 1:**
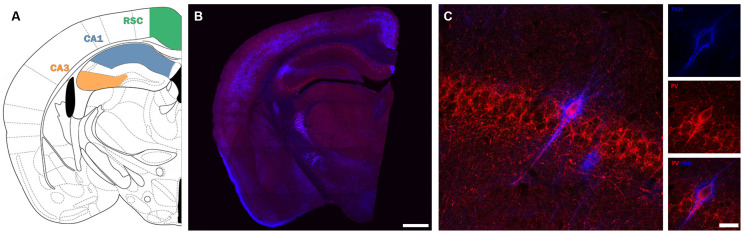
Description of the regions and cells under study. **(A)** Scheme depicting the location of the different areas under study (modified from Paxinos and Franklin, 2008) **(B)** Panoramic confocal microphotograph showing the general distribution of PV+ interneurons (red) and PNNs (blue) in a representative section coinciding with the scheme shown in **(A)**. **(C)** High magnification confocal images from CA1 *stratum pyramidale*, showing a PV-immunoreactive neuron surrounded by PNNs. Scale bars: 500 μm **(B)**, 20 μm **(C)**.

To study the number of PV immunoreactive neurons and of PNNs and their co-localization in the hippocampal regions and the RSC (Figure 1), we used a modified version of the fractionator method (West, 1993; Nacher et al., 2002). We counted all labeled cells within each section, covering 100% of the sample area in the different subdivisions and layers. The fractionator sampling scheme refers to examining one out of every six 50 μm-thick randomly selected sections. The images used for the analysis covered all the area of the region under analysis (CA3, CA1 or retrosplenial cortex), were obtained with a confocal microscope (Olympus FV-10) using a 10 × objective. They were 2D projections of confocal stacks covering all the thickness of the sections. The images were processed afterward using FIJI/ ImageJ software (Schindelin et al., 2012). All the slides were coded before the quantification, and the code was not broken until the end of the analysis.

### Statistics

After checking the homoscedasticity by Levene’s test, and the normality of the data *via* the Shapiro-Wilk Test, we performed 2-way ANOVAs considering treatment (Vehicle and MK801) and rearing (Social and Isolation) as between-subjects factors. Outliers that we excluded using Grubbs’ Test. For the ANOVAs in which we obtained a significant interaction factor, we performed *post hoc* analyses by using the Bonferroni test. We performed statistics using the number of animals as the sample number (n) and expressed values as mean ± standard error of the mean (SEM). We set the cutoff for statistical significance as *p* = 0.05. For all analyses and data presentation, we used GraphPad Prism 9 Software.

## Results

### Effects of the Double Hit Model on the Number of PV Expressing Neurons and PNNs in the Hippocampal CA1 Region

**Figure 2:**
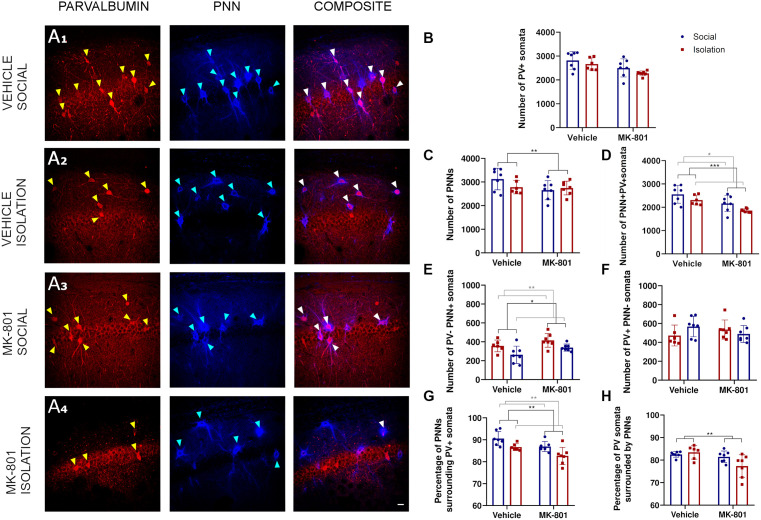
Analysis of parvalbumin (PV) expressing cells and perineuronal nets (PNNs) in the CA1 region of the hippocampus. **(A1–A4)** Confocal images from the CA1 showing PV expressing neurons **(red)**, PNNs **(blue)**, and their co-localization (PNN+ PV+). **(B–F)**Histograms indicating changes in the total numbers of PV+ somata **(B)**, PNNs **(C)**, PV+ cells surrounded by PNNs **(D)**, PNNs not surrounding PV^+^ cells **(E)**, and PV+ cells not surrounded by PNNs **(F)**. **(G,H)** Histograms indicating the percentages of PNNs surrounding PV+ somata **(G)** and PV+ somata surrounded by PNNs **(H)**. *p < 0.05; **p < 0.01; ***p < 0.001. Scale bar: 20 μm.

Our analysis in the CA1 (Figure 2A) did not reveal a significant interaction between treatment (MK801 vs. Vehicle) and rearing (Social vs. Isolation) for any parameter analyzed. Consequently, *post hoc* comparisons were not applicable (Table 1). No differences were observed in the number of PV+ cells (Figure 2B). However, the ANOVA showed an effect of the treatment on the total number of PNNs (Figure 2C, *p* = 0.0059; F_1, 23_ = 9.212). We observed significant effect of treatment (Figure 2D, *p* = 0.0009; F_1, 23_ = 14.62). and rearing (Figure 2D, *p* = 0.0200; F_1, 23_ = 6.246) on the total number of PV expressing neurons surrounded by PNNs. We observed a significant decrease of these parameters in the animals that received MK801. The ANOVA also revealed an effect of rearing (Figure 2E, *p* = 0.0038; F_1, 23_ = 10.38) and treatment (Figure 2E, *p* = 0.0183; F_1, 23_ = 6, 447) in the number of PNNs not surrounding PV+ cells: we noticed a significant decrease in the group of isolated animals.

**Table 1:** Summary of results in the hippocampal regions CA1 and CA3.

	Hippocampus CA1		
	Source of variation (two-way ANOVA)		
Total number	Treatment	Rearing	Interaction
PV+PNN+	*p* = 0.0009 *F*_(1,23)_ = 14.62	*p* = 0.0200 *F*_(1,23)_ = 6.246	*p* = 0.7973 *F*_(1,23)_ = 0.6749
PV+ somata	*p* = 0.2872 *F*_(1,23)_ = 3.190	*p* = 0.3888 *F*_(1,23)_ = 0.7716	*p* = 0.1423 *F*_(1,23)_ = 2.308
PNNs	*p* = 0.0059 *F*_(1,23)_ = 9.212	*p* = 0.1175 *F*_(1,23)_ = 2.646	*p* = 0.7510 *F*_(1,23)_ = 0.1031
PV-PNN+	*p* = 0.0183 *F*_(1,23)_ = 6.447	*p* = 0.0038 *F*_(1,23)_ = 10.38	*p* = 0.7440 *F*_(1,23)_ = 0.1092
PV+PNN-	*p* = 0.8413 *F*_(1,23)_ = 0.4099	*p* = 0.5425 *F*_(1,23)_ = 0.3822	*p* = 0.861 *F*_(1,23)_ = 3.216
**Percentage**
PNNs surrounding PV+ somata	*p* = 0.0027*F*_(1,23)_ = 11.33	*p* = 0.0016*F*_(1,23)_ = 12.87	*p* = 0.8664*F*_head(1,23)_ = 0.0289
PV+ somata surrounded by PNNs	*p* = 0.0091*F*_(1,23)_ = 8.124	*p* = 0.2161*F*_(1,23)_ = 1.618	*p* = 0.581*F*_(1,23)_ = 3.976
	**Hippocampus CA3**		
	**Source of variation (two-way ANOVA)**		
**Total number**	**Treatment**	**Rearing**	**Interaction**
PV+PNN+	*p* = 0.4845 *F*_(1,23)_ = 0.5049	*p* = 0.5180 *F*_(1,23)_ = 4.310	*p* = 0.180 *F*_(1,23)_ = 3.339
PV+ somata	*p* = 0.6034 *F*_(1,23)_ = 0.2775	*p* = 0.1358 *F*_(1,23)_ = 2.390	*p* = 0.167 *F*_(1,23)_ = 3.670
PNNs	*p* = 0.6190 *F*_(1,23)_ = 0.254	*p* = 0.4704 *F*_(1,23)_ = 0.5387	*p* = 0.1208 *F*_(1,23)_ = 2.596
PV-PNN+	*p* = 0.2087 *F*_(1,23)_ = 1.673	*p* = 0.3058 *F*_(1,23)_ = 1.097	*p* = 0.4957 *F*_(1,23)_ = 0.4791
PV+PNN-	*p* = 0.5531 *F*_(1, 22)_ = 0.3623	*p* = 0.8176 *F*_(1, 22)_ = 0.0544	*p* = 0.1001 *F*_(1, 22)_ = 2.936
**Percentage**
PNNs surrounding PV+ somata	*p* = 0.0383 *F*_(1,23)_ = 4.832	*p* = 0.4847 *F*_(1,23)_ = 0.5045	*p* = 0.1063 *F*_(1,23)_ = 2.825
PV+ somata surrounded by PNNs	*p* = 0.1194 *F*_(1, 21)_ = 2.626	*p* = 0.3832 *F*_(1, 21)_ = 0.7916	*p* = 0.4919 *F*_(1, 21)_ = 0.4885

*As a consequence of non-significant interaction between treatment and rearing for any parameter analyzed in the CA1 and CA3 region of the hippocampus, *post hoc* comparisons were not applicable. Thus, the table presents the statistically significant effect (two-way ANOVA) as F and *p* values*.

No differences were observed in the number of PV cells not surrounded by PNNs (Figure 2F). We also observed the effects of treatment (Figure 2G, *p* = 0.0027; F_1, 23_ = 11.33) and rearing (Figure 2G, *p* = 0.0016; F_1, 23_ = 12.87) on the percentage of PNNs surrounding PV+ cells, and of treatment on the percentage of PV positive cells that were surrounded by PNNs (Figure 2H, *p* = 0.0091; F_1, 23_ = 8.124).

### Effects of the Double Hit Model on the Number of PV Expressing Neurons and PNNs in the Hippocampal CA3 Region

**Figure 3:**
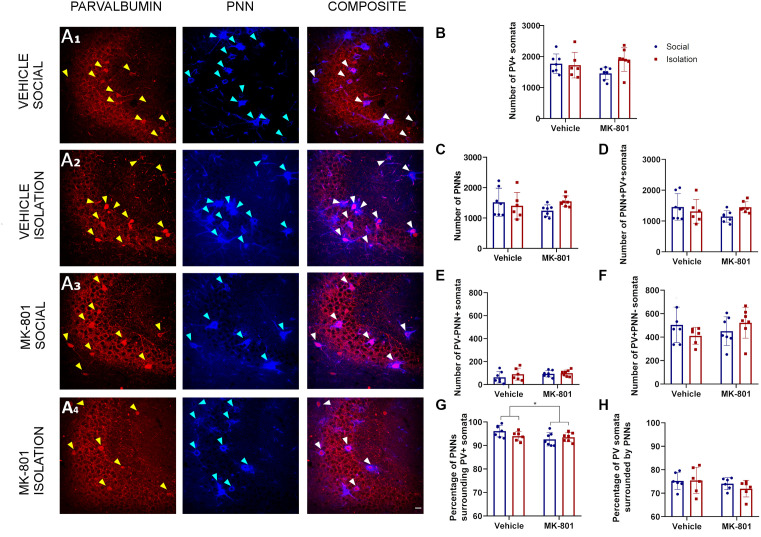
Analysis of parvalbumin (PV) expressing cells and perineuronal nets (PNNs) in the CA3 region of the hippocampus. **(A1–A4)** Confocal images from the CA3, showing PV expressing neurons **(red)**, PNNs **(blue)**, and their co-localization (PNN+PV+). B-F: Histograms indicating changes in the total numbers of PV+ somata **(B)**, PNNs **(C)**, PV+ cells surrounded by PNNs **(D)**, PNNs not surrounding PV^+^ cells **(E)**, and PV+ cells not surrounded by PNNs **(F)**. G&H: Histograms indicating the percentages of PNNs surrounding PV+ somata **(G)** and PV+ somata surrounded by PNNs **(H)**. *p < 0.05. Scale bar: 20 μm.

Our analysis in the CA3 (Figure 3A) did not reveal a significant interaction between treatment (MK801 vs. Vehicle) and rearing (Social vs. Isolation) for any parameter analyzed. Consequently, *post hoc* comparisons were not applicable (Table 1). The number of PV+ cells, PNNs, and PV+ cells surrounded by PNNs were not affected by any experimental condition (Table 1; Figures 3B–D). Similarly, we did not observe any significant differences in the number of PNNs not surrounding PV+ cells (Table 1; Figure 3E) and in the number of PV+ cells not surrounded by PNNs (Table 1; Figure 3F). However, the treatment affected the percentage of PNNs surrounding PV+ somata (Figure 3G, *p* = 0.0383; F_1, 23_ = 4.832). We did not observe any significant difference in the percentage of PV+ cells surrounded by PNNs (Figure 3H).

### Effects of the Double Hit Model on the Number of PV Expressing Neurons and PNNs in the Retrosplenial Cortex

**Figure 4:**
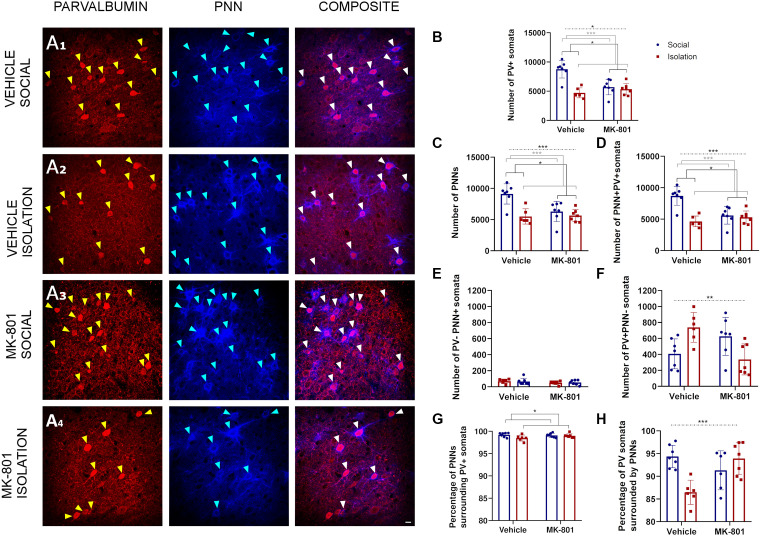
Analysis of parvalbumin (PV) expressing cells and perineuronal nets (PNNs) in the retrosplenial cortex. **(A1–A4)** Confocal images from the retrosplenial cortex, showing PV expressing neurons **(red)**, PNNs **(blue)**, and their co-localization (PNN+ PV+). **(B–F)** Histograms indicating changes in the total numbers of PV+ somata **(B)**, PNNs **(C)**, PV+ cells surrounded by PNNs **(D)**, PNNs not surrounding PV^+^ cells **(E)**, and PV+ cells not surrounded by PNNs **(F)**. **(G,H)** Histograms indicating the percentages of PNNs surrounding PV+ somata **(G)** and PV+ somata surrounded by PNNs **(H)**. Dashed lines indicate significant interactions and the presence of significant differences after *post hoc* analysis. *p < 0.05; **p < 0.01; ***p < 0.001. Scale bar: 20 μm.

Our analyses in the RSC (Figure 4A) with a 2-way ANOVA allowed us to observe significant effects of treatment (MK801 vs. Vehicle) in the total number of PV+ somata (Figure 4B, *p* = 0.021; F_1, 23_ = 6.141), PNNs (Figure 4C, *p* = 0.0157; F_1, 23_ = 6.803), and PV+PNN+ cells (Figure 4D, *p* = 0.0168; F_1, 23_ = 6.651). We also observed significant effects of rearing (Social vs. Isolation) in the total number of PV+ somata (Figure 4B, *p* = 0.0006; F_1, 23_ = 15.71), PNNs (Figure 4C, *p* = 0.0001; F_1, 23_ = 20.96), and PV+PNN+ cells (Figure 4D, *p* = 0.0001; F_1, 23_ = 21.07). The interaction of treatment and rearing was also significant for the total number of PV+ somata (Figure 3B, *p* = 0.0117; F_1, 23_ = 7.509), PNNs (Figure 4C, *p* = 0.0006; F_1, 23_ = 15.62), and PV+PNN+ cells (Figure 4D, *p* = 0.0006; F_1, 23_ = 15.77); consequently, here *post hoc* comparisons were applicable (Table 2). In the DHM, we observed a significantly lower number of PV+ somata (Figure 4B, *p* = 0.0006), PNNs (Figure 4C, *p* = 0.0002) and PV+PNN+ cells (Figure 4D, *p* = 0.0002) when compared to control mice. Similar significant decreases were observed for the groups of animals that were just isolated or treated with MK801 (Table 2; Figures 4B–D). None of the groups showed significant changes in the number of PNNs not surrounding PV+ cells (Figure 4E). The number of PV+ neurons not surrounded by PNNs was affected by the interaction of both factors (Figure 4F, *p* = 0.0022; F_1, 23_ = 11.89), and significantly increased was observed in the group of isolated mice that received vehicle solution compared to the control group (Figure 4F, *p* = 0.0457). However, DHM animals did not show a significant change in this parameter. The percentage of PNNs surrounding PV+ somata was significantly affected by rearing (Figure 4G, *p* = 0.0482; F_1, 23_ = 4.354), but not by treatment or the interaction. However, the interaction of both factors affected the percentage of PV+ cells surrounded by PNNs (Figure 4H, *p* = 0.0006; F_1, 22_ = 16.28); a significant decrease was observed in the isolated animals that received vehicle solution (Figure 4H, *p* = 0.0018), and in the socially reared animals treated with MK801 (Figure 4H, *p* = 0.014), and when compared to the control group. However, no significant differences for this later parameter were observed between control and DHM mice.

**Table 2:** Summary of results in the RSC.

	Retrosplenial cortex					
	Source of Variation (two-way ANOVA)			Group differences (from Veh-Social)		
Total number	Treatment	Rearing	Interaction	Veh-Isolation	MK801-Social	MK801-Isolation
PV+PNN+	*p* = 0.0168 *F*_(1,23)_ = 6.651	*p* = 0.0001 *F*_(1,23)_ = 21.07	*p* = 0.0006 *F*_(1,23)_ = 15.77	****↓ *p* < 0.0001	***↓ *p* = 0.0006	***↓ *p* = 0.0002
PV+ somata	*p* = 0.0210 *F*_(1,23)_ = 6.141	*p* = 0.0006 *F*_(1,23)_ = 15.71	*p* = 0.0117 *F*_(1,23)_ = 7.509	***↓ *p* = 0.0006	**↓ *p* = 0.0052	***↓ *p* = 0.0006
PNNs	*p* = 0.0157 *F*_(1,23)_ = 6.803	*p* = 0.0001 *F*_(1,23)_ = 20.96	*p* = 0.0006 *F*_(1,23)_ = 15.62	****↓ *p* < 0.0001	*** *p* = 0.0005	*** *p* = 0.0002
PV-PNN+	*p* = 0.1972 *F*_(1,23)_ = 1.764	*p* = 0.9809 *F*_(1,23)_ = 0.5031	*p* = 0.7564 *F*_(1,23)_ = 0.0985	n/a	n/a	n/a
PV+PNN-	*p* = 0.1792 *F*_(1,23)_ = 1.919	*p* = 0.4730 *F*_(1,23)_ = 0.5324	*p* = 0.0022 *F*_(1,23)_ = 11.89	*↑*p* = 0.0457	ns*p* = 0.4601	ns*p* = 0.9642
**Percentage**						
PNNs surrounding PV+ somata	*p* = 0.3425 *F*_(1,23)_ = 0.9396	*p* = 0.0482 *F*_(1,23)_ = 4.354	*p* = 0.1249 *F*_(1,23)_ = 2.536	n/a	n/a	n/a
PV+ somata surrounded by PNNs	*p* = 0.1054 *F*_(1, 22)_ = 2.852	*p* = 0.114 *F*_(1, 22)_ = 2.943	*p* = 0.0006 *F*_(1, 22)_ = 16.28	**↓ *p* = 0.0018	*↓ *p* = 0.014	ns*p* = 0.8857

In the cases, where the interaction between treatment and rearing were significant, *post hoc* comparisons were applicable, and the direction and difference from Veh-Social (control) are marked with symbols: (↓) decrease, (↑) increase; *p < 0.05; **p < 0.01; ***p < 0.001; ****p < 0.0001, no significant effect (ns). When there was a non-significant interaction between treatment and rearing, *post hoc* comparisons were not applicable (n/a).

## Discussion

In the present study, we have used a DHM, combining a perinatal MK801 injection to disturb early postnatal development and post-weaning social isolation as an early life aversive experience. We have investigated the effect of the model and each of its factors on the subpopulation of PV expressing interneurons and PNNs in the hippocampus and the RSC of adult male mice. Both regions were affected by isolation and MK801 treatment, although the effect of the DHM was **particularly** evident in the RSC, where the interaction of both interventions was significant. In the following paragraphs, we discuss these results, comparing them with the previous literature from animal models and postmortem studies on schizophrenic patients.

It is important to note that in the present study, we estimated the total numbers of cells. The main advantage of using the fractionator method and such stereological estimation is that it allows to obtain a high sensitivity of counting (Golub et al., 2015). In our previous studies, we have shown that it is a powerful tool for quantitative analysis (Gilabert-Juan et al., 2013; Castillo-Gómez et al., 2017). This is in contrast with most of the studies cited here, which are mainly based on the analysis of densities.

In the CA3, we did not observe any significant differences in the number of PV+ cells after MK801 treatment or social isolation. Our results are in accordance with studies that also failed to detect significant differences in the number of PV+ cells in the CA3 or the dentate gyrus of mice treated with the NMDA receptor antagonist ketamine (Koh et al., 2016). However, in this report, the treatment was chronic for one month during adolescence. Other studies found conflicting results in adult rats but also used chronic treatments: significant decreases in the number of PV+ neurons in CA3 after treatment with ketamine for five consecutive days (Keilhoff et al., 2004; Nullmeier et al., 2011). Moreover, another study showed an age dependent effect, finding decreases in PV+ cell density in all subregions of the hippocampus after chronic ketamine administration in 1-month-old rats, while observing a modest increase in 6 months old animals (Honeycutt and Chrobak, 2018). Similarly, chronic ketamine treatment in adult rats increased the number of PV+ interneurons in CA3 located outside the *stratum oriens*, and an overall numerical increase in PV+ cells in this region (Sabbagh et al., 2013). It has to be noted that, although ketamine is also an NMDA receptor antagonist, the effects of chronic ketamine administration might be very different from the ones that we observed in our model. Most of these chronic treatments are applied during the adolescence or in adult animals. Moreover, the persistent blockade of the receptors should also lead to alterations different to those observed with the single perinatal dose used in our model. Our intention is to cause a slight perturbation in the stages of development that are occurring at this time (Makinodan et al., 2012). Most likely we will have alterations in last phases of migration of certain neurons, neurite extension and synaptogenesis. We do not think that the propsychotic effects of the drug (at this time and at this dose) might be relevant (compared to its effects on neurodevelopment). It is also worth mentioning that we study the animals when they are adults, 83 days after the administration of the drug.

In the CA1, no differences were observed in the number of PV+ cells after treatment, isolation, or in the DHM. In line with our present results, our previous study in an identical DHM in rats did not show significant changes in this parameter in all the different hippocampal subfields or when analyzing this structure as a whole; we also did not detect changes in PV mRNA expression (Gilabert-Juan et al., 2013). Similar negative results have been found when studying the number of PV+ cells in the hippocampus of rats exposed to another NMDAR antagonist, phencyclidine, during early postnatal life (Kaalund et al., 2013). By contrast, mice exposed chronically to ketamine during adolescence had a significantly lower number of PV-immunoreactive neurons in the CA1 of the hippocampus (Koh et al., 2016). Recently, also Fujikawa and coworkers found that the density of PV+ neurons was decreased in the CA1, but not in the CA2/3 region of mice treated subchronically with ketamine, although the age of drug administration and sacrifice of the animals were not indicated (Fujikawa et al., 2020).

Studies using postweaning social isolation in rats have rendered different results regarding the number or density of PV+ interneurons. In the hippocampus, some studies in male rats did not find alterations in these parameters or in PV mRNA expression (Kaalund et al., 2013), while another study in mice only found reductions in the dentate gyrus (Ueno et al., 2017a). However, some others found reductions in the density of PV+ cells in CA2/3, but not in CA1, in female rats (Harte et al., 2007) and both in CA1 and CA2/3 in male rats (Powell et al., 2015). Alterations in PV+ interneurons and PNNs have also been observed after juvenile social isolation and maternal separation and/or multiple hits of early life adversity in the PFC of rodents (Bicks et al., 2020; Gildawie et al., 2021).

Schizophrenic patients show deficits in the relative density of PV immunoreactive neurons in the hippocampus. Interestingly, a study found that these reductions occurred in all hippocampal subfields, were more apparent in male than in female patients, and was unrelated to age or duration of illness (Zhang and Reynolds, 2002). Similar results were found by Konradi (2011) and coworkers, who found that the number of PV+ cells, as well as the level of PV mRNA expression, were reduced when considering the whole hippocampus. However, significant reductions in PV+ neurons were only found in CA1 but not in CA2/CA3 regions (Konradi et al., 2011). It is tempting to conclude that, at least in terms of hippocampal PV+ cells, our DHM is not a good model of schizophrenia, if we compare it with the reductions observed in human patients, because there is a lack of interaction between factors in this region. Even in the RSC where both factors interact with each other, the DHM does not lead to stronger effects than the single factors. However, it has to be noted that in the CA1 there is a non-significant reduction in the number of PV+ cells and a significant decrease in the number of PV+ cells surrounded by PNN. We believe that the DHM is still valid because in it the effects of the 2 factors are present (although independently). We have previously shown that other behavioral and neurochemical (in the PFC and the amygdala) changes relevant for schizophrenia are present in the DHM, sometimes contributed by one of the factors alone and sometimes through the interaction of both factors (Castillo-Gómez et al., 2017; Garcia-Mompo et al., 2020).

Regarding the RSC, we observed significant alterations induced by housing and treatment and the interaction of both factors in the total number of PV expressing interneurons. The most remarkable decrease was observed in the group of isolated animals. To our knowledge, this is the first report on such alterations in the RSC in an animal model of schizophrenia. However, a previous report described that a single dose of phencyclidine in adult rats increased the number of PV+ interneurons displaying an immunoreactive c-Fos nucleus in the RSC (Hervig et al., 2016). Notwithstanding, there are several differences between the two models. The most important discrepancy in our opinion is the age of the animals, while in our model the NMDA antagonist injection occurred during the last stages of development, in the (Hervig et al., 2016) study the animals were adults. Consequently, the drug should perturb few, if any, developmental processes, and it is likely that the phencyclidine induces plasticity in already mature circuits. Hervig et al. (2016) focused only on active PV+ cells (identified by c-Fos nuclear expression) while we counted every PV+ cell in the RSC. Finally, it is also possible that the use of a different NMDA antagonist and of a different rodent species also contribute to the different outcomes. In any case, similar decreases in the number of PV+ cells to the ones observed in our study were detected in the infralimbic cortex in an identical DHM in rats (Gilabert-Juan et al., 2013) and mice (Castillo-Gómez et al., 2017). Although there are no reports on alterations of PV+ neurons in the RSC of schizophrenic patients, the decrease of PV+ cells observed in the RSC of our DHM is in line with some *postmortem* studies in the PFC (Reynolds et al., 2001; Beasley et al., 2002). However, contrary to these results, other studies did not find significant differences in the density of PV expressing interneurons **in the PFC** (Hashimoto et al., 2003; Enwright et al., 2016; Alcaide et al., 2019).

Interestingly, the RSC is one of the first presenting degenerative changes in animal models of psychosis following the injection of low doses of NMDA receptor antagonists (Olney et al., 1991; Olney and Farber, 1995). The reduction in the number of PV expressing interneurons observed in the RSC may be related to the extensive cell death caused by the perinatal NMDA antagonist administration during perinatal development (Powell et al., 2012). However, in most of the previous models using perinatal NMDA receptor antagonist administration the drugs were delivered repeatedly and we used only a low single dose, which should not produce high levels of cell death. It should be noted that the decrease in PV+ cells could also be due to a reduction in the expression of PV and not related to any neurodegenerative event. Future studies using molecular analyses to evaluate the levels of expression of PV protein and mRNA, or the analysis of different time points after NMDA receptor administration, should help to understand the reduction in PV immunoreactive neurons.

Another parameter that we have studied is the presence of PNNs and their co-localization with PV+ interneurons. The effect of MK801 injection and the animal isolation was particularly notable in the CA1. In this hippocampal region, we observed an effect of treatment on the total number of PNNs, and a significant impact of both treatment and isolation on the percentage of PNNs surrounding PV+ cells. In all the cases, the effects lead to decreases in the number of structures. Ueno et al. (2017a) did not find significant changes in the density of PNNs in any hippocampal subfield, but, similar to us, reported reductions in PV+PNN+ neurons in the CA1 and dentate gyrus of mice subjected to social isolation after weaning. It must be noted, however, that in this study, the animals were sacrificed at P56 and not at P90 as in our study. Our results are also similar to those reported in the CA1 of mice subchronically treated with ketamine, in which the density of PNN and PV+ neurons surrounded by PNNs was lower than in vehicle-treated mice (Fujikawa et al., 2020).

Interestingly, the number of PNNs not surrounding PV+ cells was significantly affected by isolation and by treatment. It should be noted that most, if not all, these cells had the morphology of pyramidal neurons. Therefore, it is possible that a small subpopulation of excitatory neurons, especially in the hippocampus, may be also surrounded by PNNs. The presence of PNNs surrounding excitatory neurons has already been suggested in the PFC of humans (Enwright et al., 2016; Alcaide et al., 2019). It should also be noted that in CA1 the proportion of PNNs not surrounding PV+ cells and PV+ cells not surrounded by PNN is very low (approximately 10% and 20%, respectively). Similar low proportions can be found in the other regions analyzed. Unfortunately, very little is known about the role of PNNs in principal neurons.

In the RSC, we reported an effect of treatment, isolation, and the interaction of both factors on the number of PNNs and PV+ cells surrounded by PNNs. We observed a significant reduction in DHM animals, as well as in just isolated or treated with MK801 mice in comparison to control animals. The percentage of PNNs surrounding PV+ cells was significantly affected by isolation itself. Interestingly, in this neocortical region, the proportion of PNNs not surrounding PV+ cells and PV+ cells not surrounded by PNN in control animals was even lower than in CA1 (0% and 5%, respectively). There are only two reports describing the density and distribution of PNNs in the RSC of adult and aging mice (Ueno et al., 2018, 2019), and to our knowledge, this is the first report describing alterations in PNNs or PV+ cells in an animal model of early adverse experiences and/or neurodevelopmental alterations in early life in this region. However, there is evidence that exposure to ethanol at P7 reduces the density of PV+ cells and PNNs in the RSC (Lewin et al., 2018). Our results also align with previous ones in similar rodent models, which have found reductions in the number of PNNs in other neocortical regions, particularly the PFC, in mice and rats (Paylor et al., 2016; Castillo-Gómez et al., 2017; Matuszko et al., 2017; Gildawie et al., 2021). Interestingly, a recent study from our laboratory, which used the same DHM, also found a significant decrease in the density of PNNs and PV expressing interneurons surrounded by PNNs in the prelimbic and infralimbic cortices (Garcia-Mompo et al., 2020).

Although studies on the impact of schizophrenia on PV+ cells or PNN in the RSC are still lacking, similar results to those seen in animal models have been found in different neocortical regions, including the PFC and the superior temporal cortex (Pantazopoulos et al., 2010, 2015; Mauney et al., 2013; Berretta et al., 2015). A recent report from our laboratory did not find reductions in the dorsolateral PFC in PNNs in schizophrenic patients. However, this reduction reached significance in bipolar disorder patients and when considering all patients (bipolar and schizophrenic) who displayed psychosis (Alcaide et al., 2019).

Despite the little knowledge existent on the involvement of the RSC in schizophrenia, it appears to be an interesting candidate for investigating its etiopathology. There are previous findings of abnormal connectivity of this cortical region in the default mode network of patients (Bluhm et al., 2009). Abnormal activity in a semantic memory task (Tendolkar et al., 2004), as well as and in memory encoding (Hofer et al., 2003), has also been observed in this region. Connectivity differences in patients with schizophrenia were also observed between the RSC and regions of the brain related to language processing, suggesting its role in the etiology of auditory hallucinations (Bluhm et al., 2009). The RSC is closely interconnected with the hippocampus and the anterior thalamic nuclei (van Groen and Wyss, 1992). Indeed, the alteration of the RSC connectivity disrupts the brain circuit that connects the hippocampus with the thalamus (Sharp et al., 2001).

Since both the hippocampus and the RSC are implicated in spatial memory processing, it is possible that the effects of social isolation may be due not only to the lack of social interaction but to the fact that the “isolation” cages were smaller (half the surface) than the “group” ones. However, one has to take also into account that the surface available per animal in the “group” cages was half of that in the “isolation” cages.

The reductions in PNNs density/number may be a generalized feature in the brain of schizophrenic patients since they also have been observed in extracortical structures, such as the amygdala, the olfactory epithelium, or the thalamic reticular nucleus (Pantazopoulos et al., 2010, 2013; Steullet et al., 2018). This idea is in accordance with recent findings from genetic studies showing that several genes encode PNNs components and enzymes involved in ECM remodeling, present altered expression in schizophrenia (Buxbaum et al., 2008; Rybakowski et al., 2009; Dow et al., 2011; Groszewska et al., 2011; Ripke, 2014). Furthermore, deletion of the gene encoding collagen XIX in mice (*col19a1^−/−^)* which are associated with familial schizophrenia, results in the reduction of PNNs in the hippocampus (Su et al., 2017). Since PNNs appear to have a protective role against oxidative stress in PV expressing cells (Cabungcal et al., 2013), the decrease in PNNs number may render the PV+ cells more vulnerable to the deleterious effects of free radicals and result in cellular degeneration, which has been described in schizophrenic patients (Andreazza et al., 2008; Yao and Keshavan, 2011). The loss of PNNs may induce changes in the connectivity of PV expressing interneurons, which in turn could destabilize the inhibitory input of these cells on principal neurons and the excitation/inhibition (E/I) balance. A recent study from our laboratory, has shown that PNN depletion has a profound impact in the connectivity and function of PV+ interneurons, at least in the PFC (Carceller et al., 2020). Interestingly, this **E/I** imbalance has been proposed to underlie the etiopathology of schizophrenia (Lewis et al., 2012; Inan et al., 2013; Sun et al., 2013; Morishita et al., 2015).

In future studies it would be interesting to measure in more detail parameters associated to the structure and composition of PNN, such as the intensity of fluorescence of the *Wisteria floribunda* agglutinin, the expression of different proteoglycans and associated proteins or the fine structure of the mesh and its holes. Animals exposed to post-weaning social isolation displayed reduced PNN intensity in the PFC (Ueno et al., 2017a,b). Moreover, maternal separation was found to affect the structural integrity of PNNs and PV neurons in the PFC in a sex-dependent manner (Gildawie et al., 2020).

Because of the crucial role of PV+ interneurons have in regulating the activity of pyramidal neurons and the excitation/inhibition balance (Ferguson and Gao, 2018), and the influence of PNNs on their connectivity and plasticity (Testa et al., 2019; Wingert and Sorg, 2021); the alterations in PV+ and PNNs in both the RSC and the hippocampus and their impact on the connectivity of these regions deserve further studies in schizophrenic patients and animal models of the disease.

## Data Availability Statement

The raw data supporting the conclusions of this article will be made available by the authors, without undue reservation.

## Ethics Statement

The animal study was reviewed and approved by Committee on Bioethics of the Universitat de València.

## Author Contributions

JN and EC-G designed the experiment. PK, AR, YG, MP-R, and MB performed the experiments and quantifications. PK and JN wrote the manuscript and all the authors revised and edited the final version. All authors contributed to the article and approved the submitted version.

## Conflict of Interest

The authors declare that the research was conducted in the absence of any commercial or financial relationships that could be construed as a potential conflict of interest.

## Publisher’s Note

All claims expressed in this article are solely those of the authors and do not necessarily represent those of their affiliated organizations, or those of the publisher, the editors and the reviewers. Any product that may be evaluated in this article, or claim that may be made by its manufacturer, is not guaranteed or endorsed by the publisher.
